# Periarticular Embolization as an Alternative Treatment for Surgery-Ineligible Patients with Hip Osteoarthritis: A Prospective Comparative Study

**DOI:** 10.3390/jcm15135108

**Published:** 2026-07-01

**Authors:** Andrei Marian Feier, Florin Bloj, Octav Marius Russu, Andrei Bloj, Rares Nechifor, Tudor Sorin Pop

**Affiliations:** 1 Department M4 Clinical Sciences, Orthopedics and Traumatology I, George Emil Palade University of Medicine, Pharmacy, Science, and Technology of Targu Mures, 540139 Targu Mures, Romania; andrei.feier@umfst.ro (A.M.F.);; 2 Department of Orthopaedics and Traumatology, Clinical County Hospital of Mures, 540139 Targu Mures, Romania; 3 Memorial Baneasa Hospital, 013812 Bucharest, Romania; 4 Ponderas Academic Hospital, 021659 Bucharest, Romania; office@drnechifor.ro; 5 Department 8, Radiology, Medical Imaging, and Interventional Radiology II, Carol Davila University of Medicine and Pharmacy, 030167 Bucharest, Romania

**Keywords:** hip osteoarthritis, transarterial periarticular embolization, conservative care

## Abstract

**Background/Objective**: Hip osteoarthritis (HOA) is a major source of pain and disability worldwide. Although total hip arthroplasty (THA) provides substantial symptomatic improvement, a subgroup of patients remains ineligible because of severe comorbidities, frailty or elevated perioperative risk. Conservative treatments provide only temporary symptom control and transarterial periarticular embolization (TAPE) has emerged as a minimally invasive intervention targeting abnormal periarticular hypervascularity and inflammation. The aim was to compare clinical outcomes of TAPE and conservative care (CC) in patients with symptomatic HOA considered unsuitable for THA. **Methods**: A prospective non-randomized two-centre cohort study included consecutive adults aged ≥60 years with symptomatic HOA and baseline visual analogue scale (VAS) pain scores over 40. Patients were managed with either TAPE or structured CC. The primary endpoint was change in VAS pain score from baseline to 6 months. Secondary outcomes included Lower Extremity Functional Scale (LEFS), Timed Up-and-Go (TUG) and analgesic use. Patients were evaluated at baseline, 1, 3 and 6 months. **Results**: A total of 81 patients were screened, 69 were enrolled and 62 were included in the complete case longitudinal analysis. Baseline symptom severity was greater in the TAPE group, with higher VAS scores (73.6 ± 12.5 vs. 63.7 ± 14.1; *p* = 0.003) and lower-joint space width (1.37 ± 0.79 vs. 2.07 ± 0.89 mm; *p* < 0.001). The reduction in pain occurred during the first three months following embolization, after which symptom trajectories stabilized. Mean VAS pain in the TAPE group decreased from 73.6 ± 12.5 at baseline to 55.4 ± 13.0 at three months and 56.8 ± 13.6 at six months. LEFS improved in both groups throughout follow-up. **Conclusions**: TAPE was associated with symptom improvement and short-term safety in a small cohort of surgery-ineligible patients with HOA. The observed benefits appeared greatest within the first three months.

## 1. Introduction

Hip osteoarthritis (HOA) is a common and disabling musculoskeletal disorder, with a reported global prevalence of approximately 8.6% when defined using Kellgren–Lawrence grade 2 or higher, and a higher estimated prevalence in Europe of approximately 12.6% [1]. Its burden continues to rise as populations age, obesity becomes more frequent, and survival with chronic disease increases, creating a larger group of patients exposed to long-term mechanical, metabolic, and inflammatory risk factors [2]. A high body mass index remains one of the most relevant modifiable contributors to osteoarthritis, accounting for more than 20% of osteoarthritis cases worldwide [2]. Beyond pain and structural joint damage, HOA produces a marked loss in health-related quality of life, with utility decrements of approximately 0.3–0.35 compared with the general population, a reduction greater than that reported for several other chronic diseases [3]. The condition also increases healthcare use, reduces mobility and productivity, and has been associated with anxiety, depression and higher mortality in older adults compared with age-matched controls [4]. Total hip arthroplasty (THA) remains the definitive treatment for advanced symptomatic HOA when pain, functional limitation, and radiographic disease persist despite appropriate nonoperative care [5]. Contemporary THA provides reliable pain relief and functional recovery, with approximately 90% of recipients reporting little or no residual pain and around 80% of implants remaining in situ at 20 years [5]. A substantial proportion of patients do not meet these conditions because active infection, uncontrolled systemic disease, severe cardiopulmonary risk, poor metabolic control, morbid obesity, active tobacco use, frailty, advanced cognitive impairment, or inability to complete rehabilitation may make surgery unsafe or inappropriate [6,7]. Strict eligibility thresholds restrict access to care for patients with complex medical or socioeconomic profiles, and rigid cutoffs worsen existing disparities in arthroplasty delivery [6]. Surgery-ineligible patients with symptomatic HOA represent a clinically difficult group because they often have the highest symptom burden and the fewest durable treatment options [8]. Uncontrolled cardiovascular disease, decompensated heart failure, recent myocardial infarction, morbid obesity, diabetes mellitus, renal failure and severe cognitive impairment each increase perioperative risk beyond an acceptable level [9,10]. These patients remain symptomatic for prolonged periods because they are too high-risk for THA yet too advanced in disease stage to obtain sustained benefit from standard conservative measures [5,6]. This creates a therapeutic gap between palliative symptom control and definitive joint replacement, leaving many patients with persistent pain, functional loss, and reduced independence [3]. Conservative care (CC) combines education, activity modification, exercise therapy, strengthening programmes, weight loss when appropriate and pharmacological treatment [11]. Nonsteroidal anti-inflammatory drugs carry gastrointestinal, renal and cardiovascular risks in older adults and patients with comorbidities [6,12]. Most nonoperative therapies do not modify the underlying disease process, and many patients with advanced HOA still require arthroplasty for sustained symptom control [6,13]. Transarterial periarticular embolization (TAPE) has emerged as a minimally invasive procedure aimed at reducing pain related to abnormal periarticular neovascularity and synovial inflammation in osteoarthritis [14]. The biological reasoning is based on the association between chronic joint pain, inflammatory synovial activity and pathological hypervascular changes around the affected joint, which provide a target for selective arterial embolization [15]. For patients who cannot undergo THA, this technique offers a joint-preserving palliative option. The lack of comparative evidence is relevant because surgery-ineligible patients are treated with repeated cycles of conservative therapy despite limited durability and no established disease-modifying effect [16]. Therefore, the purpose of the present study was to compare the clinical outcomes of TAPE and structured CC in surgery-ineligible patients with symptomatic HOA.

## 2. Materials and Methods

### 2.1. Study Design

This was a prospective comparative cohort study conducted in adults with symptomatic HOA who were considered unsuitable for THA. The study protocol was approved by the institutional ethics committee (approval no. 470, date of 13 October 2025), and all participants provided written informed consent before enrolment. The study was conducted in collaboration between the Interventional Radiology Unit of Ares Excellence Center, Memorial Băneasa Hospital, Bucharest, Romania, and the Department of Orthopaedics and Traumatology, Clinical County Hospital of Mureș, Târgu Mureș, Romania.

### 2.2. Participants and Treatment Allocation

We recruited consecutive adults aged 60 years or older with radiographic HOA graded using the Tönnis classification (grade II and III) and a baseline pain score of at least 40 on a 0–100 visual analogue scale (VAS). All included patients were ineligible for THA due to high associated medical risk. All patients needed to be able to attend scheduled visits and to complete the required outcome measures. Patients were excluded if they had a history of inflammatory arthritis, avascular necrosis of the femoral head, prior fractures of the hip/pelvis, or any active hip infection. Additional exclusion criteria included prior hip arthroplasty on the affected side or contralateral side, any intra-articular corticosteroid or hyaluronic acid injection within the previous three months, and known allergies to iodinated contrast agents. Patients were managed according to one of two treatment pathways: transarterial periarticular embolization (TAPE group) or structured conservative care (CC group). Eligibility for TAPE or CC was assessed before recruitment began. Patients were offered either TAPE or structured CC according to previous treatment history. Patients who had not completed at least two OARSI-recommended conservative treatments, or who had contraindications to angiographic procedures, were managed with the structured CC protocol [11]. The reason for treatment pathway selection was recorded.

### 2.3. Transarterial Embolization Procedure

**Figure 1:**
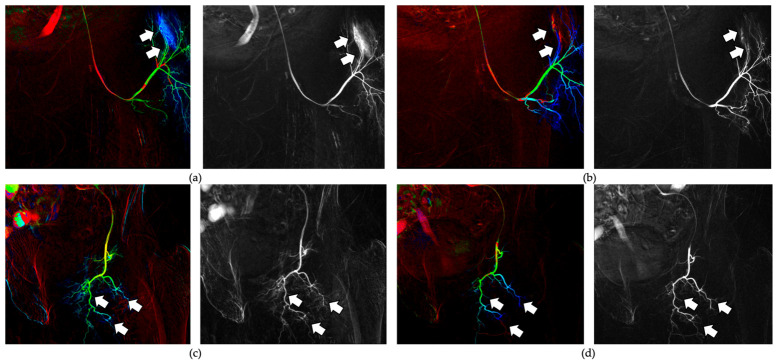
Representative digital subtraction angiography images (Kinepict Medical Imaging Tool; Kinepict Health Ltd., Budapest, Hungary) obtained during transarterial periarticular embolization. (**a**) Selective angiography demonstrating pathological periarticular hypervascularity blush (arrows) arising from superior periarticular branches of lateral circumflex femoral artery. (**b**) Post-embolization angiography showing reduction in the abnormal vascular blush following selective embolization while preserving parent vessel patency. (**c**) Selective angiography of obturator artery branches demonstrating abnormal periarticular neovascularity and synovial hypervascularity surrounding the hip joint (arrows). (**d**) Final angiographic control after embolization demonstrating reduction in pathological blush with preservation of normal distal arterial flow.

TAPE was performed by the same interventional radiologist with more than five years of experience in musculoskeletal embolization (F.B.). Procedures were conducted under local anesthesia using left brachial arterial access. A 4 Fr, 125 cm multipurpose (Radifocus Optitorque; Terumo Corporation, Tokyo, Japan) catheter was advanced under fluoroscopic guidance to selectively catheterize arterial branches. Selective angiography was performed to identify abnormal periarticular hypervascularity and pathological vascular blush. A 0.021-inch microcatheter (Direxion; Boston Scientific, Marlborough, MA, USA) was subsequently used for superselective catheterization of target vessels, including branches of the inferior gluteal artery, obturator artery and lateral circumflex femoral artery when indicated according to angiographic blush. The embolic suspension consisted of imipenem/cilastatin sodium (IPM/CS; 500 mg/500 mg) mixed with contrast medium containing iodixanol (Visipaque^®^; GE Healthcare Limited, Chalfont St. Giles, UK). The embolic suspension was continuously agitated throughout delivery to avoid sedimentation. Embolization was performed under real-time fluoroscopic guidance using slow injections in 0.2–0.3 mL aliquots until near stasis and reduction in abnormal distal vascularity were achieved, while preserving flow within the parent vessel and avoiding reflux into non-target branches. Fluoroscopic blank roadmap imaging was used during embolization. Technical success was defined as reduction or disappearance of abnormal vascular blush (Figure 1) on final angiography. The extent of baseline periarticular hypervascularity was assessed qualitatively during the procedure.

At the end of the procedure, a control angiogram was performed and hemostasis was achieved by manual compression of the brachial access site. Radiation exposure (dose-area product) and contrast volume were documented for each procedure. Patients remained under observation for at least four hours following intervention, including monitoring of vital signs, access site status and neurovascular examination.

### 2.4. Conservative Care Protocol

Patients allocated to the structured CC group followed a predefined non-surgical protocol based on OARSI recommendations for HOA [11]. The protocol included three mandatory core components: arthritis education, structured land-based exercise, and analgesics. These were applied uniformly to all patients.

#### 2.4.1. Arthritis Education Protocol

At baseline, all patients received standardized education regarding HOA, activity, avoidance of symptom-aggravating overload, use of walking aids when needed and the importance of maintaining regular low-impact physical activity. Patients with body mass index ≥ 25 kg/m^2^ received written advice on weight reduction.

#### 2.4.2. Exercise Protocol

The exercise component consisted of a home-based exercise programme prescribed for 12 weeks [17]. Patients were instructed to perform the programme at least three times per week. The programme included hip range of motion exercises, isometric and isotonic strengthening of the gluteal and periarticular hip muscles, quadriceps strengthening, stretching of the hip flexors and adductors and aerobic stationary cycling. Exercise intensity was adapted only according to pain response.

#### 2.4.3. Pharmacological Protocol

Pharmacological treatment followed a stepwise protocol. Patients were prescribed celecoxib 200 mg once daily for 10 days with associated esomeprazole 20 mg gastroprotection. The prescription was repeated once every 4 weeks. Acetaminophen was permitted as analgesia during the 12-week period. Intra-articular corticosteroid injection was not used as part of the routine conservative protocol. Hyaluronic acid injections, platelet-rich plasma and other intra-articular biological treatments were not permitted during follow-up.

Adherence to the conservative protocol, medication use and any protocol deviations were prospectively recorded at each follow-up visit.

### 2.5. Outcomes and Measurements

All patient-reported outcomes were collected using paper or electronic forms administered by trained staff (A.B) who were not involved in treatment decisions. The VAS was administered as a 100 mm horizontal line with verbal anchors at each end (“no pain” and “worst pain imaginable”) and the Lower Extremity Functional Scale (LEFS) was administered in its standard 20 item format without modification (0–80 item scale). MCID response was defined as a reduction of at least 15 points in VAS pain from baseline. The Timed Up-and-Go (TUG) test measured the time required to stand up from a standard chair, walk 3 metres, turn, walk back and sit down. The same staff, environment and instructions were used for both groups. Weight-bearing anteroposterior pelvis and axial hip radiographs were obtained at our centre within 3 months before the index date. Two senior physicians, blinded to treatment allocation and clinical data, independently graded osteoarthritis using the Tönnis classification and measured joint space width. The index date was defined as the date of the embolization procedure for the TAPE group and the date of initiation of the CC protocol for the CC group. Patients were evaluated at baseline, 1 month, 3 months, and 6 months. The primary endpoint was the change in pain, measured using the VAS from baseline to 6 months. Secondary endpoint included changes in LEFS from baseline to 6 months and analysis of TUG test.

### 2.6. Statistical Analysis

Continuous variables were reported as means with standard deviations or medians with interquartile ranges, depending on distribution. Categorical variables were presented as counts and percentages. Given the non-randomized design, a propensity score for receiving embolization was constructed using baseline variables, including age, sex, body mass index, Tönnis grade, baseline pain, comorbidity index and allocation clinical factors. Overlap weighting was applied to reduce baseline imbalances between groups. The primary outcome was analyzed using a weighted linear mixed-effects model with fixed effects for treatment group, time, and group by time interaction and a random intercept for each patient. Results were reported as adjusted mean differences with 95% confidence intervals. The primary analysis was performed on the complete-case longitudinal cohort. Patients with incomplete longitudinal follow-up after treatment allocation were excluded from the continuous outcome analysis. A two-sided *p*-value < 0.05 was considered statistically significant. The planned sample size was based on the primary endpoint, defined as the difference in change in VAS pain score from baseline to 6 months. With a minimal clinically important difference (MCID) of 15 points reported in the literature, a standard deviation of 20 points, a two-sided alpha level of 0.05, and 80% power, approximately 28 patients per group were required [18]. All eligible TAPE patients were included, and a larger CC group was recruited to improve precision and support propensity score adjustment. Software analysis was performed using IBM SPSS Statistics for Windows (v28.3; IBM Corp., Armonk, NY, USA).

## 3. Results

**Figure 2:**
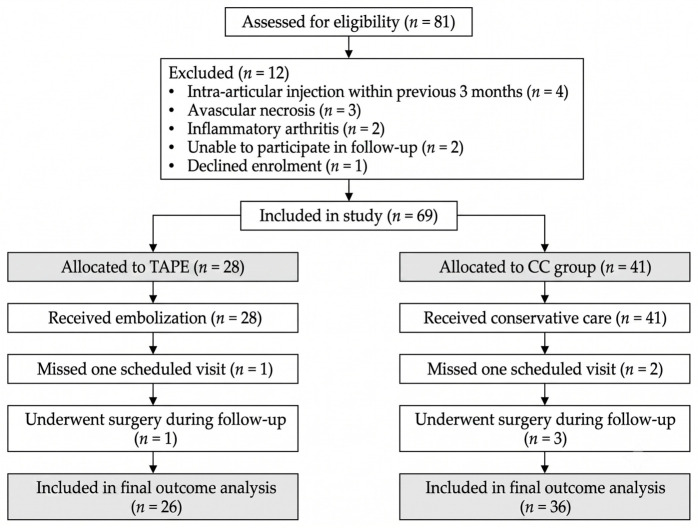
Flow diagram of patient screening, exclusions, treatment allocation, follow-up and analysis.

A total of 81 consecutive patients with symptomatic HOA were screened. Twelve patients were excluded before enrolment: four had received intra-articular injections within the previous three months, three had avascular necrosis, two had inflammatory arthritis, two were unable to participate in follow-up, and one declined enrolment. Therefore, 69 patients were enrolled in the study, comprising 28 patients in the TAPE group and 41 patients in the structured conservative care group. During follow-up, three patients missed one scheduled visit and four patients underwent surgery unrelated to the index HOA diagnosis. These seven patients were excluded from continuous longitudinal outcome analysis according to protocol. The final analyzed cohort included 62 patients: 26 in the TAPE group and 36 in the conservative care group. The participant flow diagram is illustrated in Figure 2.

Baseline characteristics are presented in Table 1. Patients managed with TAPE were slightly younger and had greater baseline symptom severity. The mean age was 66.3 ± 7.6 years in the TAPE group and 67.0 ± 8.0 years in the CC group (*p* = 0.637).

Baseline pain scores were significantly higher in the TAPE group (73.6 ± 12.5 vs. 63.7 ± 14.1; *p* = 0.003) (Table 2). Patients undergoing TAPE demonstrated higher comorbidity burden and more severe radiographic disease, with lower-joint space width and a larger proportion of Tönnis III OA.

The greatest reduction in pain occurred during the first three months following TAPE, after which symptom trajectories stabilized. The mean VAS pain score in the TAPE group decreased from 73.6 ± 12.5 at baseline to 62.1 ± 18.4 at one month, 55.4 ± 13.0 at three months, and 56.8 ± 13.6 at six months. In the CC group, the mean VAS pain score decreased from 63.7 ± 14.1 at baseline to 59.6 ± 16.5 at one month, 53.8 ± 15.9 at three months, and 55.4 ± 16.4 at six months. Weighted overlap mixed-effects analysis demonstrated a greater reduction in pain among patients treated with TAPE, with an adjusted mean difference at six months of −9.1 points (95% CI −12.8 to −5.5; *p* < 0.001) (Table 3). Although between-group difference was statistically significant, its magnitude was below the predefined 15-point threshold used to define an MCID in VAS pain. Emergency surgery unrelated to main diagnosis was performed in one patient (3.6%) in the TAPE group and three patients (7.3%) in the CC group, who were removed for continuous longitudinal outcomes.

**Figure 3:**
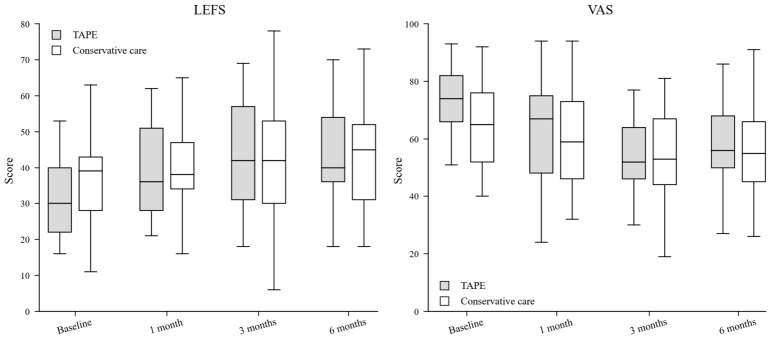
Longitudinal changes in LEFS and VAS scores according to treatment group.

LEFS scores increased by 11.6 points in the TAPE group and 6.3 points in the CC group between baseline and six months (Figure 3).

Patients treated with TAPE had greater improvement in mobility measures. Improvements in TUG were slightly higher in the TAPE group. At six months, 71% of patients in the TAPE group achieved the predefined MCID for pain improvement compared with 34% in the CC group (RR 2.09; 95% CI 1.2–3.7). Adherence to the structured conservative care protocol was satisfactory. Thirty-five patients (85.4%) reported that they completed the prescribed exercise programme at least three times weekly during the first 12 weeks, while six patients reported irregular participation because of pain exacerbation and/or limited mobility. Educational counselling was completed for all patients at baseline. Mean weekly analgesic exposure progressively decreased during follow-up in both groups, with a greater reduction observed after embolization. No intra-articular injections, biological therapies or opioid use were reported. In the TAPE group, all procedures were technically successful and no repeat embolization procedures were required.

Adverse events were infrequent and no major complications occurred. In the TAPE group, transient post-procedural pain and minor access site hematomas were observed and resolved without intervention. In the CC group, mild gastrointestinal symptoms related to analgesic use were reported. No neurovascular complications, infections, or embolization-related serious adverse events occurred.

## 4. Discussion

This prospective comparative study found that TAPE was associated with greater short-term improvement in pain-related outcomes, lower-limb function, mobility, and analgesic use than structured conservative care in surgery-ineligible patients with symptomatic hip osteoarthritis. However, the magnitude of the adjusted between-group difference in VAS pain at six months was 9.1 points and therefore remained below the predefined 15-point MCID threshold. The MCID analysis adds a clinical perspective. Although the adjusted mean between-group difference in VAS pain did not reach the predefined 15-point MCID threshold, a higher proportion of patients treated with TAPE achieved an individual meaningful pain reduction compared with structured CC. This suggests that the average treatment effect underestimates the perceived benefit in a subset of individuals. The clinical response was most evident during the first three months after embolization and then stabilized with an early symptom-modifying effect and not progressive improvement. The present findings should be interpreted as preliminary evidence that TAPE provides clinically relevant benefits in selected patients and not definitive proof of superior efficacy over structured conservative care. These findings support further evaluation of TAPE as a possible palliative option for selected high-risk patients with symptomatic HOA who remain unsuitable for THA. The results align with the small hip TAPE literature, although direct evidence in HOA is limited. Early hip-specific reports have consisted mainly of proof-of-concept experience, small cohorts and single-arm feasibility studies, including embolization for hip synovitis, lateral femoral circumflex artery embolization in mixed hip pain populations and prospective single-arm work in surgery-ineligible HOA [14,19,20,21]. The present study adds a structured CC comparator, which is uncommon in the published hip literature. The embolization literature is still led by genicular artery embolization for knee osteoarthritis where prospective cohorts and randomized studies have shown symptom improvement [22,23,24]. For this reason, the knee literature supports the biological and clinical plausibility of the present findings but it cannot be used as direct proof of efficacy in HOA. The mechanism underlying symptom improvement after TAPE was not directly measured in this study. The procedure was based on angiographic identification and embolization of abnormal periarticular hypervascularity [25]. In osteoarthritis, synovitis, inflammatory angiogenesis, neovessel formation, and sensory nerve growth contribute to pain generation and create a reason for reducing abnormal vascular supply [25]. Knee embolization studies have reported angiographic neovessels and MRI changes consistent with reduced synovial inflammatory activity after embolization and preclinical studies support the concept that embolization of abnormal neovessels can alter inflammatory vascularity [26,27]. Experimental support for this vascular mechanism comes from the swine arthritis model reported by Kamisako et al., in which transcatheter embolization of abnormal neovessels modified inflammatory joint vascularity, although these findings remain preclinical [28]. In the present cohort, the early pain and functional response is consistent with this model, but no direct mechanistic conclusion can be drawn. No imaging follow-up was performed to evaluate biological response after embolization. Although TAPE was guided by angiographic identification of abnormal periarticular vascular blush, this study did not include post-procedural MRI, contrast-enhanced MRI, Doppler ultrasound, or other perfusion-based imaging to assess changes in synovitis or pathological neovascularity. The biological rationale for TAPE in HOA should be interpreted differently from genicular artery embolization in knee osteoarthritis. The hip has a smaller and less-accessible synovial compartment than the knee and pain in HOA is multifactorial, involving the following: structural degeneration, joint space narrowing, subchondral bone changes, capsular irritation, labral or chondral pathology, and periarticular soft-tissue pain generators. Given this combination of factors, the clinical relevance of synovial inflammation or hypervascularity may be less predictable in HOA than in knee osteoarthritis. The intended target of TAPE is not the synovium alone but angiographically abnormal periarticular hypervascularity and neovascularity in symptomatic vascular territories. Recent hip-specific embolization studies have described abnormal vascular blush or tortuous neovessels arising from the lateral and medial circumflex femoral arteries as well as gluteal and obturator branches, with pain distribution sometimes corresponding to these vascular territories [29,30]. This hypothesis supports a more selective neurovascular rationale in which abnormal neovessels may accompany or stimulate pain-sensitive nerve fibres and contribute to regional pain sensitization. Accordingly, TAPE should be interpreted as a potential palliative option for selected surgery-ineligible patients with demonstrable periarticular hypervascularity and not a general treatment or solution for all patients with HOA.

The choice of IPM/CS as the embolic agent deserves consideration as IPM/CS acts as a temporary crystalline particulate embolic agent and has been used in musculoskeletal embolization because it allows relatively rapid recanalization, reducing concern for persistent non-target ischemia [31]. IPM/CS particles are predominantly small and major or complete recanalization can occur within 48 h, unlike permanent microspheres, which remain occlusive for longer [31]. This temporary profile is relevant around the hip, where prolonged ischemia to periarticular, muscular, capsular or osseous branches raises theoretical safety concerns. However, transient embolization does not necessarily preclude sustained clinical benefit. In musculoskeletal embolization, symptom improvement after IPM/CS has been reported beyond the expected embolic residence time and the therapeutic effect relates to interruption of the hypervascular inflammatory and neoangiogenic pain component not to permanent devascularization [32]. In the present cohort, the early improvement during the first three months followed by stabilization reflect attenuation of the hyperemic or synovitic pain component, while further improvement remained limited by the persistent structural substrate of HOA. Permanent calibrated particles theoretically provide more durable vessel occlusion, but current comparative evidence, mainly from genicular artery embolization, has not established clear superiority over IPM/CS and should be weighed against the possibility of more persistent ischemic adverse events [33,34]. No major embolization-related complications were observed in the present cohort. However, the sample size and follow-up duration remain insufficient to draw definitive conclusions regarding procedural safety, rare adverse events or long-term vascular effects. Future studies should aim to standardize angiographic assessment of periarticular hypervascularity in HOA. At present, no validated hip angiographic grading system for pathological vascular blush is established. Prospective grading of baseline blush severity, ideally combined with perfusion imaging or post-embolization imaging biomarkers, could help determine whether the extent of neovascularity correlates with clinical response after TAPE. Such standardization would improve patient selection, procedural reporting, and comparison across future studies. The observed events in the TAPE group were limited to transient post-procedural pain and minor access site hematomas that resolved without intervention. This pattern is consistent with early hip-specific embolization reports, in which major complications were uncommon. Similar safety findings have also been reported in the broader genicular artery embolization literature for knee osteoarthritis, although these data should be considered supportive evidence for musculoskeletal embolization and not direct evidence for HOA as available hip cohorts are small and insufficient to define rare adverse events [19,24,25,28,35,36]. The hip has specific safety concerns because of its arterial anatomy and the need to avoid non-target embolization to branches of bone, skin, muscle, or neural structures. Larger cohorts with longer surveillance are still required to define the risk of rare events such as femoral head osteonecrosis, skin ischemia, persistent neuropathy, contrast-related complications, and radiation-related concerns [19]. For patients with symptomatic HOA who are unsuitable for total hip arthroplasty because of age, comorbidity, frailty, or patient preference, several minimally invasive options may be considered after failure of structured conservative care, including image-guided intra-articular injections, pericapsular nerve blocks, transarterial periarticular embolization, and radiofrequency ablation (RFA) of the hip articular branches. Hip RFA is increasingly used as a palliative option for patients awaiting, avoiding or deemed ineligible for arthroplasty [37]. Published studies have evaluated pulsed, conventional thermal and cooled RFA, most commonly targeting the articular branches of the femoral and obturator nerves under fluoroscopic guidance with or without ultrasound. Although the evidence base is more heterogeneous than that for knee RFA, multiple cohorts and more recent comparative studies suggest that hip RFA can provide clinically meaningful pain relief and parallel functional improvement for approximately 3–12 months, with serious complications reported infrequently [38,39,40]. HOA-focused studies have shown improvement in HOOS, WOMAC, OHS, HHS, and physical quality-of-life measures and recent active-comparator studies suggest that RFA may be at least competitive with intra-articular corticosteroid injection and offer greater durability beyond the first month [4]. TAPE and RFA should probably not be viewed as purely competing interventions, because they target different components of hip OA pain biology. RFA primarily interrupts or modulates nociceptive afferent transmission from the hip capsule through the articular branches of the femoral and obturator nerves, whereas TAPE is intended to reduce abnormal periarticular neovascularization, synovial hypervascularity, and the accompanying inflammatory vascular supply that may contribute to pain sensitization [41,42]. RFA is best understood as a denervation-based analgesic strategy, while TAPE is a vascular-inflammatory mechanism-targeted strategy. Cross-study comparison remains indirect, but contemporary hip RFA studies have reported meaningful short- to mid-term pain relief with functional gains and feasible repeat denervation, whereas recent hip embolization cohorts have reported sustained improvement in pain and HOOS-based function at 12 months, low early conversion to arthroplasty, and few serious adverse events [43,44,45]. These techniques prove complementary and not mutually exclusive. Future studies could test phenotype-guided or sequential strategies, for example reserving TAPE for patients with imaging or clinical evidence of active synovitis and hypervascularity, RFA for patients with a predominantly nociceptive phenotype or strong response to diagnostic articular-branch blockade, or combined/sequential approaches for mixed phenotypes. At present, however, such strategies require prospective comparative evaluation.

The present analysis relies on several strengths. This study prospectively enrolled a clinically difficult population that is often excluded from arthroplasty trials and interventional studies: patients with symptomatic HOA considered unsuitable for THA because of high associated medical and anesthesia risk. The comparator was not passive observation, but a predefined structured conservative-care protocol including education, land-based exercise, and analgesic treatment aligned with guideline-based non-surgical management [5,11,46]. Outcomes were collected at multiple time points and included pain, lower-limb function, mobility, and analgesic use.

This study also has limitations. Treatment allocation was not randomized and was influenced by previous treatment history, symptom severity, and procedural eligibility. This produced important baseline imbalances between groups. Patients treated with TAPE had higher baseline pain scores, higher comorbidity burden, more frequent walking-aid use, and narrower joint space width, suggesting a more symptomatic and structurally advanced disease profile. These differences may have influenced the observed treatment effects. Higher baseline pain may allow greater measurable improvement and may contribute to regression toward the mean, whereas more advanced radiographic disease and higher comorbidity may reduce the probability of sustained clinical response. Although overlap weighting was used to reduce measured confounding, this method cannot replace randomization and cannot account for unmeasured factors such as pain phenotype, patient expectations, frailty, inflammatory activity, rehabilitation adherence, or previous treatment exposure. Therefore, the adjusted results should be interpreted as exploratory estimates of association rather than definitive evidence of treatment efficacy. The absence of blinding and of a sham procedure are relevant because pain and function are subjective outcomes and sham-controlled knee embolization trials have produced conflicting results [22,23,24]. Seven enrolled patients were excluded from the complete-case longitudinal analysis after treatment allocation because of missed follow-up data or surgery unrelated to the index HOA diagnosis. This could have introduced attrition bias given the modest sample size. Follow-up was limited to 6 months and restricted the interpretation of durability. TAPE should not be interpreted as a disease-modifying treatment or as a definitive alternative to THA. The stabilization of symptom trajectories after the first 3 months suggests that the main effect is early symptom modulation and not sustained structural benefit. Symptom recurrence after the initial response is possible and the need for repeat embolization procedures has not been defined for HOA. Some patients eventually progress to THA if symptoms remain unacceptable or if their surgical risk profile improves. Longer follow-up is therefore needed to determine the duration of benefit, the frequency and safety of repeat procedures and the rate of eventual conversion to THA. TAPE was offered after previous conservative treatment failure; this group could have shown a higher rate of refractory disease. This design reflects real-world decision-making but limits causal interpretation. The generalizability of the results is limited by the single operator procedural technique, the use of imipenem/cilastatin only and the specific comorbid population. These findings position TAPE as a potential symptom-modifying option for patients who remain symptomatic despite structured CC and cannot safely opt for THA. This distinction is important as THA remains the definitive treatment for advanced symptomatic HOA in fit surgical candidates, and current arthroplasty-timing guidance does not support repeated delay once nonoperative therapy has failed in patients suitable for surgery [6].

## 5. Conclusions

In surgery-ineligible patients with symptomatic HOA, TAPE was associated with statistically greater short-term improvement in pain, lower-limb function, mobility, and analgesic use than structured CC, although the adjusted mean difference in VAS pain did not reach the predefined MCID threshold. For patients with advanced HOA who remain unsuitable for THA because of comorbidity, frailty, or excessive perioperative risk, TAPE offers a minimally invasive palliative option when guideline-based conservative treatment provides insufficient control. Larger multicenter studies with randomized allocation, longer surveillance, standardized embolization protocols, and imaging or biomarker assessment of synovial inflammatory activity are needed to confirm safe and optimal patient selection.

## Figures and Tables

**Table 1:** Baseline characteristics of enrolled patients.

	TAPE (*n* = 28)	CC (*n* = 41)	*p*-Value
Age, years	66.3 ± 7.6	67.0 ± 8.0	0.637
Female sex	17 (60.7%)	23 (56.0%)	0.894
BMI (kg/m^2^)	29.6 ± 3.5	29.5 ± 3.9	0.879
Charlson Comorbidity Index	3.46 ± 1.91	2.41 ± 1.43	0.017
Walking aid use	16 (57.1%)	19 (46.3%)	0.011
VAS	73.6 ± 12.5	63.7 ± 14.1	0.003
LEFS	31.5 ± 11.1	36.9 ± 12.0	0.059
Joint space width (mm)	1.37 ± 0.79	2.07 ± 0.89	<0.001

BMI—body mass index; CC—conservative care; LEFS—Lower Extremity Functional Scale; mm—millimeters; TAPE, transarterial embolization; VAS, visual analogue scale.

**Table 2:** Longitudinal outcomes in the final analyzed cohort, with values presented as mean ± standard deviation.

Outcome	Group	Baseline	1 Month	3 Months	6 Months
VAS pain	TAPE	73.6 ± 12.5	62.1 ± 18.4	55.4 ± 13.0	56.8 ± 13.6
	CC	63.7 ± 14.1	59.6 ± 16.5	53.8 ± 15.9	55.4 ± 16.4
LEFS	TAPE	31.5 ± 11.1	38.4 ± 12.3	43.7 ± 14.8	43.1 ± 13.3
	CC	36.9 ± 12.0	38.7 ± 13.2	41.4 ± 15.4	43.2 ± 13.8
TUG, s	TAPE	14.1 ± 3.5	13.0 ± 3.8	12.2 ± 4.1	12.4 ± 4.1
	CC	12.1 ± 3.0	11.8 ± 3.3	11.2 ± 3.6	11.0 ± 3.2
Analgesic DDD/week	TAPE	9.6 ± 3.2	7.7 ± 3.1	6.8 ± 3.6	6.8 ± 3.4
	CC	8.1 ± 4.3	7.4 ± 4.6	6.9 ± 4.7	6.8 ± 4.3

CC—conservative care; DDD—defined daily dose; LEFS—Lower Extremity Functional Scale; TAPE—transarterial embolization; TUG—Timed Up-and-Go; VAS—visual analogue scale.

**Table 3:** Adjusted treatment effects from overlap weighted mixed-effects analysis.

	Unadjusted Change TAPE	Unadjusted Change CC	Adjusted Difference	95% CI
VAS pain, 0–100	−18.3	−8.0	−9.1	−12.8 to −5.5
LEFS, 0–80	+12.8	+5.9	+6.9	+3.8 to +9.9
TUG, seconds	−2.0	−0.8	−1.8	−2.4 to −1.1
Analgesic DDD/week	−3.0	−1.4	−1.6	−2.2 to −1.0

CC—conservative care; CI—confidence interval; DDD—defined daily dose; LEFS—Lower Extremity Functional Scale; TAPE—transarterial periarticular embolization; TUG—Timed Up-and-Go; VAS—visual analogue scale.

## Data Availability

The data supporting the findings of this study are available from the corresponding author upon reasonable request, subject to ethical and privacy restrictions.

## Abbreviations

The following abbreviations are used in this manuscript:

BMI	Body mass index
CC	Conservative care
CI	Confidence interval
DDD	Defined daily dose
HOA	Hip osteoarthritis
IPM/CS	Imipenem/cilastatin sodium
LEFS	Lower Extremity Functional Scale
MCID	minimal clinically important difference
MRI	Magnetic resonance imaging
OA	Osteoarthritis
OARSI	Osteoarthritis Research Society International
TAPE	Transarterial periarticular embolization
THA	Total hip arthroplasty
TUG	Timed Up-and-Go
VAS	Visual analogue scale
